# Gestion des voies aériennes supérieures et cellulite cervico-faciale

**DOI:** 10.11604/pamj.2017.26.22.9090

**Published:** 2017-01-17

**Authors:** Hicham Kechna, Karim Nadour, Omar Ouzzad, Khalid Chkoura, Faical Choumi, Jaouad Loutid, Mohamed Moumine, Moulay Ahmed Hachimi

**Affiliations:** 1 Pôle d’Anesthésie Réanimation et Urgences, Hôpital Militaire Moulay Ismaïl-Meknès, Faculté de Médecine et de Pharmacie de Fès, Université Sidi Mohamed Ben Abdellah, Fès, Maroc; 2 Service Oto-Rhino Laryngologie, Hôpital Militaire Moulay Ismaïl-Meknès, Faculté de Médecine et de Pharmacie de Fès, Université Sidi Mohamed Ben Abdellah, Fès, Maroc; 3 Service de Chirurgie Maxillo Faciale, Hôpital Militaire Moulay Ismaïl-Meknès, Faculté de Médecine et de Pharmacie de Fès, Université Sidi Mohamed Ben Abdellah, Fès, Maroc

**Keywords:** Cellulite maxillo faciale, management des voies aériennes, ventilation difficile, intubation difficile, Maxillofacial cellulitis, airways management, difficult ventilation, difficult intubation

## Abstract

**Introduction:**

La cellulite maxillo faciale est urgence médico chirurgicale. Ces patients sont le plus souvent pris en charge au bloc opératoire d’une part pour la mise à plat et le drainage des collections et d’autres parts pour les prélèvements bactériologiques. L’anesthésie de ce genre de patient expose à des difficultés potentielles de contrôle des voies aériennes supérieures.

**Méthodes:**

Il s'agissait d'une étude rétrospective réalisée sur une durée de 24 mois dans le pôle d’anesthésie réanimation et urgence de l’hôpital militaire Moulay Ismail de Meknès avec la collaboration des services de stomatologie et d’oto-rhino laryngologie. On été inclus tous les patients admis au bloc pour cure chirurgicale sous anesthésie générale d’une cellulite cervicale et/ou maxillo-faciale.

**Résultats:**

Nous avons colligé 22 dossiers. Le sexe ratio était à 4,5 en faveur des hommes. L'âge moyen des patients est de 29 ans. Concernant la gestion des voies aériennes ; une intubation standard à l’aide de laryngoscope a été retenue chez la plupart des patients (17 patients). L’intubation vigile sous fibroscopie a été réalisée chez 3 patients, une trachéotomie première a été faite chez une patiente et une intubation rétrograde salvatrice a été retenue chez une autre patiente.

**Conclusion:**

Deux défis guettent tout anesthésiste prenant en charge des patients présentant une cellulite cervico-faciale, un risque de ventilation difficile et un souci d’intubation laborieuse. Les deux risques sont à envisager de principe, et où une stratégie anticipative devra être élaborée.

**Introduction:**

Maxillofacial cellulitis is a medical-surgical emergency. These patients are most often treated in the operating room on the one hand for identifying and draining the collections and on the other hand for bacteriological samples. The type of anesthetic technique used exposes to potential difficulties in controlling the upper airways.

**Methods:**

We conducted a retrospective study carried out in the intensive-care and anaesthesia units as well as in the emergency department (in collaboration with the departments of stomatology and otorhynolaryngology) at the Military Hospital Moulay Ismail, Meknès, over a period of 24 months. All patients undergoing surgery for cervical and/or maxillofacial cellulitis under general anesthesia were included in the study.

**Results:**

We collected 22 patient medical recordsd. The sex ratio was 4.5 in favour of men. The average age of our patients was 29 years. Concerning airways management; standard intubation with laryngoscope was performed in most patients (17 patients). Vigil intubation under fibroscopy was performed in 3 patients, elective tracheotomy was performed in a female patient and retrograde salvage intubation was performed in another female patient.

**Conclusion:**

Two challenges lie ahead for any anesthesiologist who provides medical care to each patient with cervicofacial cellulitis, a risk of difficult ventilation and a concern about difficult intubation. Both risks should be considered in advance and an anticipatory strategy should be developed.

## Introduction

L’anesthésie des cellulites maxillo faciales comporte une difficulté potentielle de contrôle des voies aériennes supérieures. En effet, les patients ayant une cellulite maxillo faciale ont un risque d’intubation difficile, en raison notamment d’un trismus et sont exposés au risque de ventilation au masque facial difficile en raison d’un œdème ou même d’un possible obstacle (abcès) au niveau des voies aériennes supérieures [1, 2].

## Méthodes

A travers ce travail nous rapportons l’expérience du pôle d’anesthésie réanimation et urgence de l’hôpital militaire Moulay Ismail de Meknès sur une durée de 2 ans. Il s’agit d’un travail rétrospectif en analysant les dossiers anesthésiques des différents patients admis au bloc pour cure chirurgicale sous anesthésie générale d’une cellulite cervicale et ou cervico faciale. Après une prémédication associant 0,01mg. kg-1 d´atropine et 0,1 mg.kg-1 de midazolam, administrée par voie intramusculaire une heure avant l´intervention et préparation du matériel d´intubation difficile (sondes d´intubation et lames de différentes tailles et formes, masque laryngé, fibroscope), une pré-oxygénation de 4 min était effectuée. Lors de l´induction anesthésique, la surveillance du patient était assurée par un électrocardioscope, un tensiomètre électronique et un oxymètre de pouls. Si l´intubation était prévue difficile, elle était réalisée à l´aide d´un fibroscope en ventilation spontanée; après anesthésie locale (méchage des fosses nasales par une mèche imbibée de lidocaine 5% naphazolinée, puis administration de lidocaine à 1% non adrénalinée (dose maximale = 200 mg) au niveau du hypopharynx par gargarisme) Dans les autres cas, l´intubation était réalisée sous anesthésie générale associant un hypnotique (souvent propofol (3 mg/kg), un morphinique (fentanyl 4 gamma/kg) et un curare (rocuronium 0,6mg/kg) Sur une fiche on a rempli en plus des données démographiques, la technique anesthésique choisie et le déroulement de l’induction anesthésique ainsi que l’évolution de chaque cas opéré.

## Résultats

**Figure 1:**
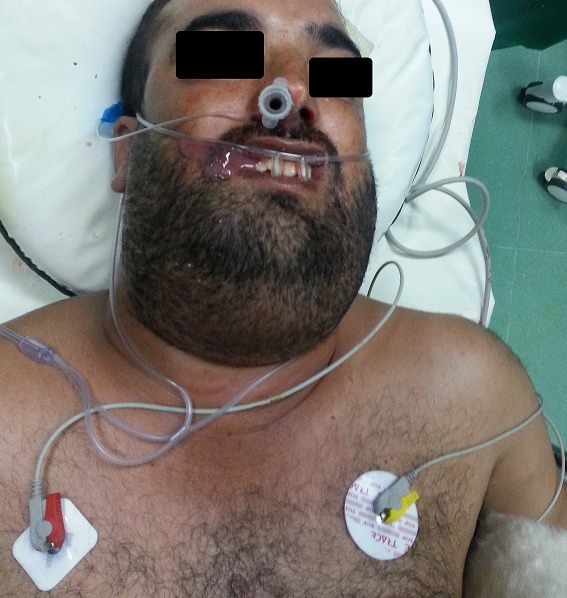
Intubation naso trachéale vigile sous fibroscopie

**Figure 2:**
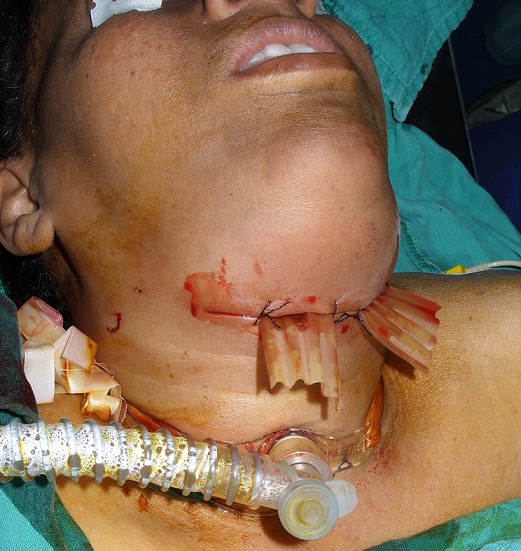
Trachéotomie première sous anesthésie locale (abcès diffus du plancher buccal, mallampati VI)

**Figure 3:**
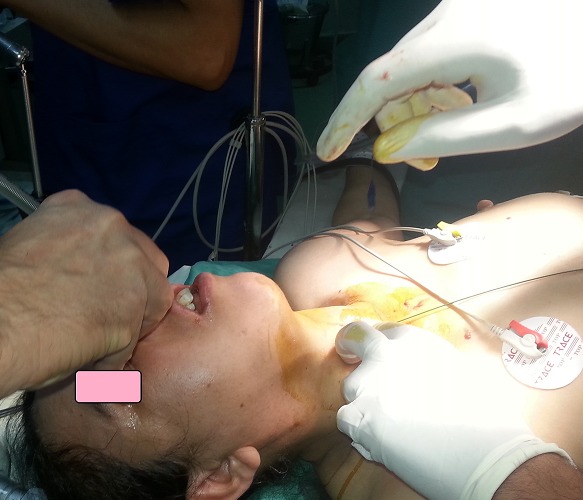
Intubation rétrograde (trismus invincible, petit abcès maxillaire)

**Figure 4:**
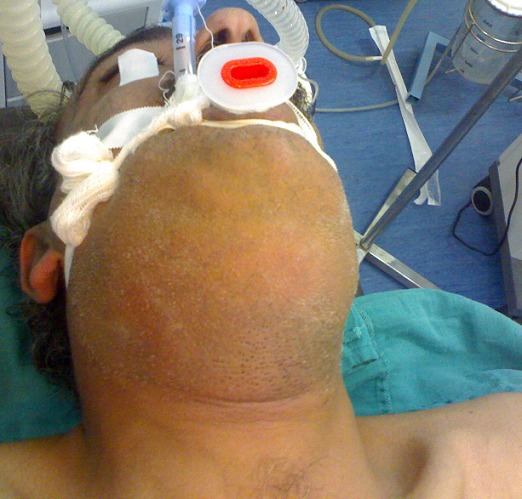
Intubation oro trachéale standard pour une cellulite débutante du plancher buccale

**Figure 5:**
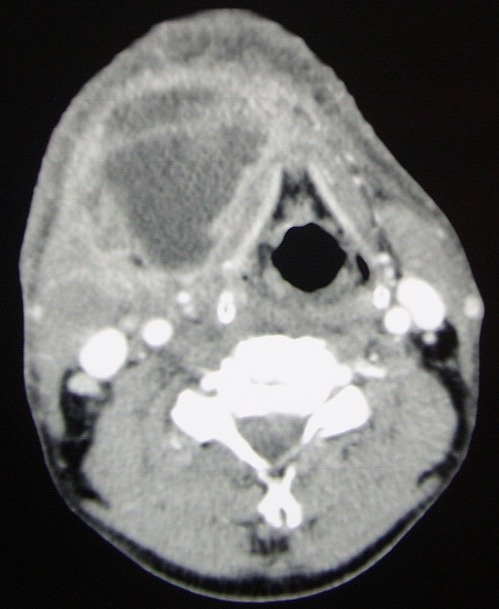
Aspects scannographiques de quelques cas colligés 1/3

**Figure 6:**
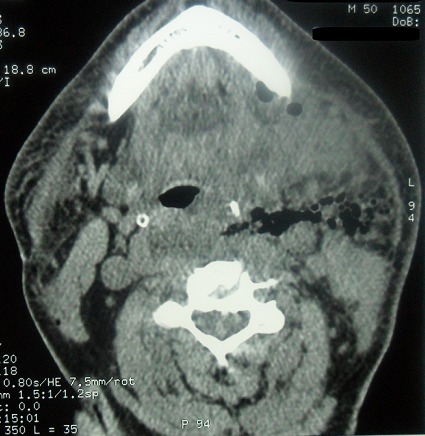
Aspects scannographiques de quelques cas colligés 2/3

**Figure 7:**
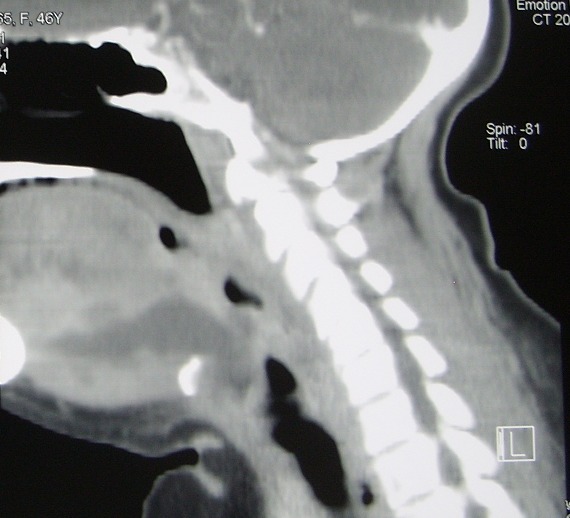
Aspects scannographiques de quelques cas colligés 3/3

On a admis au bloc opératoire 22 patients pour cellulite cervico faciale avec une nette prédominance masculine (80%). Il s’agit le plus souvent de sujet jeune (moyenne 29 ans). Le point de départ dentaire a été retenu chez la plupart de nos patients (21 patients). Concernant la gestion des voies aériennes; le recourt d’emblée à la fibroscopie avec intubation vigile a été réalisé chez 3 patients (Figure 1), une trachéotomie première (Figure 2) a été faite chez une patiente et une intubation rétrograde salvatrice (Figure 3) a été retenue chez une autre patiente ayant présenté en plus du problème de trismus invincible, une ventilation au masque difficile. Pour le reste des patients (17 patients) la gestion des voies aériennes n’a pas présenté de difficultés particulières (Figure 4). En plus de l’examen clinique et l’évaluation des différents paramètres orientant la gestion des voies aériennes en anesthésiologie (ouverture de la bouche, mallampati et la distance thyrometonnière…), les données scannographiques (Figure 5, Figure 6, Figure 7) ont été d’un apport capital dans le choix de la technique anesthésique appropriée pour chaque cas. L’évolution a été favorable pour l’ensemble de nos patients. Néanmoins, noter une desaturation postopératoire chez un patient après extubation ce qui a motivé la réalisation d’une trachéotomie du fait de la majoration des effets inflammatoires et l’obstruction des voies aériennes supérieures. Le patient a été décanulé 48 heures après.

## Discussion

Les cellulites maxillofaciales sont des affections graves nécessitant un traitement urgent et une prise en charge médicochirurgicale pluridisciplinaire. La gestion des voies aériennes représente un véritable challenge pour l’anesthésiste prenant en charge ce type de pathologie. L’usage des algorithmes élaborés dans le cadre de la réactualisation de la conférence d’experts de la Sfar en cas de difficulté du contrôle des voies aériennes est recommandé dans cette circonstance [3]. Ces algorithmes s’articulent autour de l’oxygénation du patient et des moyens mis en œuvre pour y parvenir, avec en premier lieu la possibilité d’obtenir ou non, une ventilation au masque facial efficace et en second lieu la difficulté prévue (ou non) d’intubation trachéale. Il convient que chaque centre crée son propre algorithme de prise en charge de l’intubation difficile en fonction du matériel disponible et du niveau d’apprentissage des utilisateurs potentiels. L’objectif des algorithmes de prise en charge de l’intubation difficile est la maîtrise et l’anticipation du risque. Il faut prioritairement mettre tout en œuvre pour anticiper une situation critique. Dans ce cadre, face à une intubation difficile prévue, il n’est pas recommandé de pratiquer une laryngoscopie pour évaluer la difficulté réelle de l’intubation sans avoir planifié une stratégie de prise en charge. Les différents facteurs du contrôle des voies aériennes sont pris en compte: le patient (difficulté d’oxygénation et/ou d’intubation trachéale), l’opérateur (expertise pour un panel de techniques et raisonnement par étapes) ainsi que les différentes techniques d’oxygénation et d’intubation trachéale et le matériel disponible. Le dernier élément, qui doit être déterminée est l’anesthésie qui détermine la possibilité de maintien ou non de la ventilation spontanée. L’intubation sous contrôle de la fibroscopie trachéo-bronchique est relativement rare. Toutefois, cette technique doit être maîtrisée par l’ensemble des anesthésistes réanimateurs. Même si elle est la méthode de référence, de nombreuses alternatives à la fibroscopie ont été développées et peuvent parfois être envisagées en première intention pour la prise en charge d’une intubation difficile. La vidéo-laryngoscopie pourrait constituer dans un certain nombre de situations une bonne alternative à la fibroscopie [4]. Deux facteurs rendent indispensables le recours à la fibroscopie en cas de cellulite cervicale extensive: le trismus et l’existence de collection de pus réduisant le calibre des voies aériennes. Il est indispensable avant de prendre en charge ce type de patient au bloc opératoire de tenir compte des données du scanner cervical avec injection de produit de contraste ce qui peut conduire à anticiper les difficultés qui pourra être rencontrées lors de la fibroscopie [4], et intervenir dans le choix de la technique d’anesthésique [5]. L’extubation de ces patients est un moment aussi important avec risque de desaturation par obstruction des voies aériennes. En fait l’agression chirurgicale s’ajoute aux phénomènes inflammatoires initiaux obstruant les voies aériennes supérieures. L’évaluation initiale doit prendre en considération ce risque pour prendre la décision adéquate au moment opportun. L’équipe anesthésique doit être alarmée aussi bien à l’induction qu’à l’extubation et envisager les moyens matériels et humains nécessaire pour faire face.

## Conclusion

L’anesthésie des patients présentant une cellulite cervico-faciale représente une situation où les difficultés de ventilation (donc d’oxygénation) et/ou d’intubation trachéale sont à envisager de principe, et où une stratégie anticipative devra être élaborée.

### Etat des connaissances actuelles sur le sujet

- Pathologie infectieuse touchant la région maxillo faciale d’origine dentaire le plus souvent d’où l’intérêt de la prévention par la promotion de l’hygiène bucco dentaire notamment chez les populations vulnérables;
- L’anesthésiste réanimateur est au centre de la prise en charge de cette pathologie notamment pour les patients nécessitant un drainage au bloc opératoire sous anesthésie générale comme le souligne notre travail.

### Contribution de notre étude à la connaissance

- Affection bénigne mais pouvant engager le pronostic vital à la fois par le retentissement systémique du processus infectieux que lors de la prise en charge anesthésique pour drainage chirurgicale et mise à plat (risque d’hypoxie lors de l’induction et lors du sevrage respiratoire);
- Notre étude vise à mettre en exergue le rôle anesthésique prépondérant dans la gestion de ce genre de patient;
- A travers ce travail notre équipe souligne l’intérêt d’avoir une attitude anticipative dans la gestion des voies aériennes en cas de cellulite cervico faciale et rappelle la nécessité de maitriser certains geste facile combien salvateur tel l’intubation rétrograde.
